# Supplementary material for: Transcriptomic Insights into Mechanisms of Early Seed Maturation in the Garden Pea (Pisum sativum L.)
Source: Cells. 2020 Mar 23;9(3):779. doi: 10.3390/cells9030779 (PMC7140803; doi:10.3390/cells9030779)

Zhewan−1,10(1)

Global Summary

%DE = 0.23

genes with fdr < 0.2 = 479 (217 + / 262 −)

genes with fdr < 0.1 = 395 (187 + / 208 −)

genes with fdr < 0.05 = 360 (171 + / 189 −)

genes with fdr < 0.01 = 309 (150 + / 159 −)

genes in genesets = 0

<FC> = 0

<t−score> = 0

<p−value> = 0.01

<fdr> = 0.77

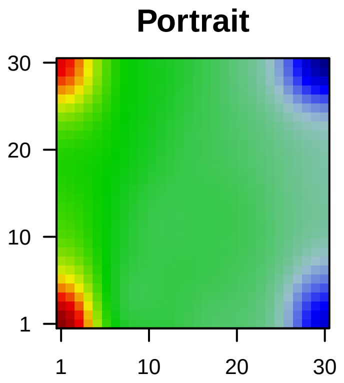

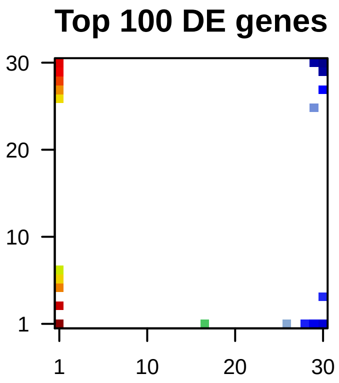

Global Genelist

Rank	ID	log(FC)	p−value	fdr	Metagene	Description
1	TRINITY_DN1	−0.72	2e−16	3e−15	30 x 1	
2	TRINITY_DN1	0.71	2e−16	3e−15	1 x 3	
3	TRINITY_DN9	−0.57	2e−16	3e−15	30 x 30	
4	TRINITY_DN7	−2.46	2e−16	3e−15	30 x 1	
5	TRINITY_DN7	2.05	2e−16	3e−15	30 x 30	
6	TRINITY_DN5	−2.39	2e−16	3e−15	30 x 30	
7	TRINITY_DN5	1.7	2e−16	3e−15	1 x 1	
8	TRINITY_DN5	1.48	2e−16	3e−15	1 x 30	
9	TRINITY_DN7	−0.64	2e−16	3e−15	30 x 30	
10	TRINITY_DN7	1.67	2e−16	3e−15	1 x 1	
11	TRINITY_DN5	−0.58	2e−16	3e−15	30 x 4	
12	TRINITY_DN9	−0.52	2e−16	3e−15	30 x 1	
13	TRINITY_DN9	−1.01	2e−16	3e−15	30 x 30	
14	TRINITY_DN9	−0.55	2e−16	3e−15	30 x 1	
15	TRINITY_DN2	0.77	2e−16	3e−15	1 x 30	
16	TRINITY_DN2	−0.53	2e−16	3e−15	29 x 30	
17	TRINITY_DN2	0.69	2e−16	3e−15	1 x 1	
18	TRINITY_DN2	−0.65	2e−16	3e−15	30 x 1	
19	TRINITY_DN2	−1.37	2e−16	3e−15	30 x 30	
20	TRINITY_DN2	1.27	2e−16	3e−15	1 x 30	

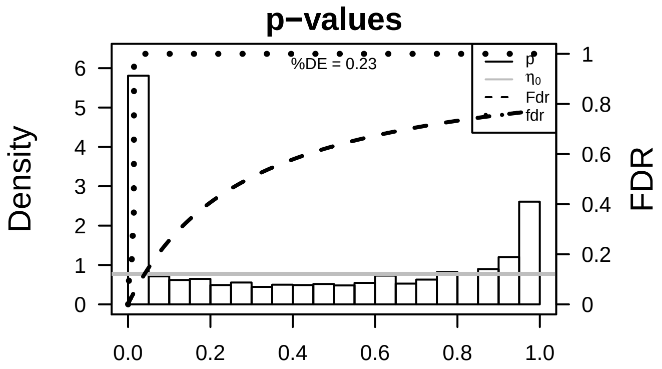

Supplement: Supplementary file 1 [file cells-09-00779-s001.zip › Supplementary materials/FileS4.zip!/FileS3/TFs - Results/Summary Sheets - Samples/Zhewan.1.10.1..pdf]
